# Silver-modified octahedral anatase particles as plasmonic photocatalyst

**DOI:** 10.1016/j.cattod.2017.05.039

**Published:** 2018-07-15

**Authors:** Z. Wei, M. Janczarek, M. Endo, C. Colbeau-Justin, B. Ohtani, E. Kowalska

**Affiliations:** a Institute for Catalysis. Hokkaido University, N21, W10, 001-0021, Sapporo, Japan; b Department of Chemical Technology, Gdansk University of Technology, Narutowicza Str. 11/12, 80-233, Gdansk, Poland; c Laboratoire de Chimie Physique, CNRS UMR 8000, Univ. Paris-Sud – Université Paris-Saclay, 91405, Orsay, France

**Keywords:** Plasmonic photocatalysts, Surface modification, Titania, Octahedral anatase particles, Photocatalytic activity, Silver NPs, AOPs, advanced oxidation processes, CDT, time needed for complete deposition of silver, DRS, diffuse reflectance spectroscopy, ETs, electron traps, HT, hydrothermal reaction, LSPR, localized surface plasmon resonance, NPs, nanoparticles, OAPs, octahedral anatase particles, SSA, specific surface area, STEM, scanning transmission electron microscopy, TNWs, potassium titanate nanowires, TRMC, time-resolved microwave conductivity, US, ultrasonication, XRD, X-ray diffraction, XPS, X-ray photoelectron spectroscopy

## Abstract

Octahedral anatase particles (OAPs) were modified with silver nanoparticles (NPs) by photodeposition method. The properties of OAPs influenced the properties of silver deposits, and thus the photocatalytic activity of the obtained silver-modified OAPs. Photocatalytic activities were tested under UV and vis irradiation for oxidative decomposition of acetic acid and oxidation of 2-propanol, respectively. The properties of silver-modified OAPs were investigated by XRD, STEM, DRS, XPS and time-resolved microwave conductivity (TRMC) method. It was found that electron traps (ETs) worked as nucleation sites for silver, resulting in formation of smaller silver NPs on smaller OAPs with larger content of ETs. The modification with silver resulted in enhanced photocatalytic activity under both UV and vis irradiation. It was found that larger crystallite size of silver NPs, and thus larger polydispersity of silver deposits resulted in broad and intense plasmon resonance peak causing enhanced visible activity. The correlation between photocatalytic activity and TRMC data, e.g., slower decay of TRMC signal for more active samples, allowed discussion on property-governed photocatalytic activities of silver-modified titania.

## Highlights

• Octahedral anatase particles (OAPs) modified with silver NPs by photodeposition.
• Ag/OAPs with enhanced photocatalytic activity under both UV and vis irradiation.
• Electron traps as nucleation sites for silver NPs.
• Polydispersity of silver NPs results in broad LSPR and thus enhanced vis activity.
• TRMC data correlate with photocatalytic activity.

## Graphical abstract

**Figure:**
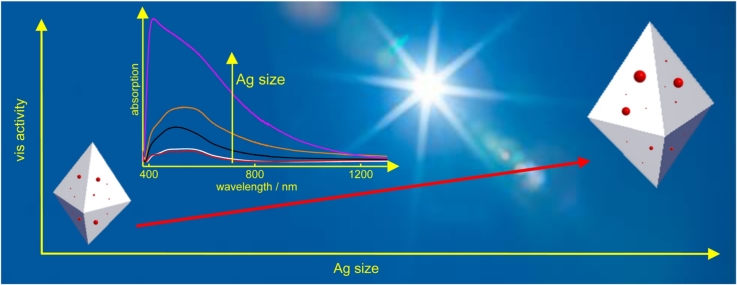

## 1 Introduction

Energy crisis and environmental pollution have become two serious challenges ahead for human beings. Solar energy has many advantages, e.g., it is a clean alternative to fossil fuels and nuclear power, cost effective, secure, eco-friendly, and especially renewable. Therefore, the effective utilization of solar energy is an advisable way to solve energy crisis. However, development of systems with high efficiency of energy conversion and energy storage is a tough task, and thus still under intensive study. It has been proposed that heterogeneous photocatalysis under solar radiation (solar photocatalysis) can be used for this purpose, as well as for environmental purification [1], [2], [3].

Titanium(IV) oxide (titania, TiO_2_) is one of the most widely investigated heterogeneous photocatalyst due to high photocatalytic activity, stability, cheapness and nontoxicity (except toxicity of nanomaterials [4]) [5], [6], [7], [8]. Moreover, commercial applications of titania have been widely reported, e.g., for self-cleaning and anti-fogging surfaces, disinfection, water purification, wastewater treatment, gas-phase purification, soil treatment, water splitting and solar energy conversion (e.g., dye sensitized solar cells) [9], [10], [11], [12], [13], [14]. However, there are two shortcomings of titania usage, i.e., (1) recombination of charge carriers (e^-^/h^+^) resulting in much lower quantum yield (typical for all semiconducting materials) than that for homogeneous photocatalysis or other advanced oxidation processes (AOPs), e.g., UV/H_2_O_2_, UV/O_3_ [15], [16], [17], [18], [19], and (2) only small part of solar energy could be efficiently used due to the wide band-gap of titania (ca. 3–3.2 eV eq. 387–413 nm) [20], [21]. Therefore, plenty of studies have been performed to improve photocatalytic performance of titania by, for example, surface modification, doping and heterojunctions with other compounds [22], [23], [24], [25], [26], [27], [28], [29], [30], as well as preparation of morphology-controlled titania nanocrystals (faceted particles) [31], [32], [33], [34], [35], [36], [37], [38]. It has been proposed that faceted particles retard the recombination of charge carriers, for instance, (i) in the case of decahedral anatase particles (DAPs) selective transfer of electrons and holes to (1 0 1) and (0 0 1) facets, respectively, was proved [35], and (ii) for octahedral anatase particles (OAPs) preferential distribution of shallow than deep electron traps (ETs) resulted in enhanced mobility of electrons [39]. Although photocatalytic activity of titania could be significantly enhanced by morphology control strategies, inactivity of titania under visible light must be overcome by other methods. Recently, modification of titania with NPs of noble metal possessing plasmon resonance feature at visible range of solar spectrum (mainly gold and silver) has been intensively investigated, and those materials are known as plasmonic photocatalysts [40], [41], [42], [43].

To obtain photoactive materials under broad range of irradiation, faceted anatase particles of octahedron shape (octahedral anatase particles, OAPs) were modified with NPs of silver and investigated in the present study. Silver (Ag) as a relative cheap noble metal has great potential for the application of plasmonic photocatalysis not only due to its plasmon resonance but also the excellent anti-microbiological (anti-fungal and anti-bacterial) properties.[44], [45], [46] In previous study on OAPs it was found that the preparation conditions had important influence on the physicochemical properties of titania (specific surface area, crystallite size, crystallinity, surface composition) including the morphology (the content of faceted NPs), and thus on the photocatalytic activity [47], [48]. To investigate how the properties of titania influence the modification with Ag, as well as how the resultant properties of Ag-modified OAPs influence the photocatalytic activity, OAPs samples varied by properties were prepared by ultrasonication-hydrothermal (US-HT) method and then modified with Ag NPs by photodeposition. It was found that an increase in the size of titania nanocrystals resulted in formation of larger silver NPs during photodeposition process. The silver properties (size and oxidation form) had significant influence on the photocatalytic activity, especially under visible light (vis) irradiation, i.e., the larger the size of silver NPs was, the higher was the vis activity.

## 2 Materials and methods

### 2.1 Preparation of bare and silver-modified OAPs

Potassium titanate nanowires (TNWs; Earthclean Tohoku Co. Ltd), synthesized by hydrothermal reaction (HT) of Evonik P25 titania (Nippon Aerosil) with potassium hydroxide (17 mol L^−1^) at 383 K for 20 h [49], were used as the precursor for preparation of bare OAPs, as reported previously [47]. In brief, 267 mg of TNWs were dispersed by ultrasonication (US) in Milli-Q water (40 mL) for 1 h, and the obtained suspension was poured in a 100-mL sealed Teflon bottle. The residue of TNWs was rinsed with an additional portion of Milli-Q water (40 mL) and added to the Teflon bottle. The bottle was inserted into an outer sleeve of a stainless autoclave and then heated in an oven at 433 K for a fixed period of time (3–24 h). The collected suspension after US-HT was dispersed by 10-min US and then centrifugally separated (12000 rpm, 20 min). The white precipitates were collected and dried overnight under vacuum (353 K, 12 h). In total, five titania samples containing OAPs were prepared by varying in duration of HT process, i.e., 3 h, 4.5 h, 6 h, 12 h and 24 h.

**Fig. 1:**
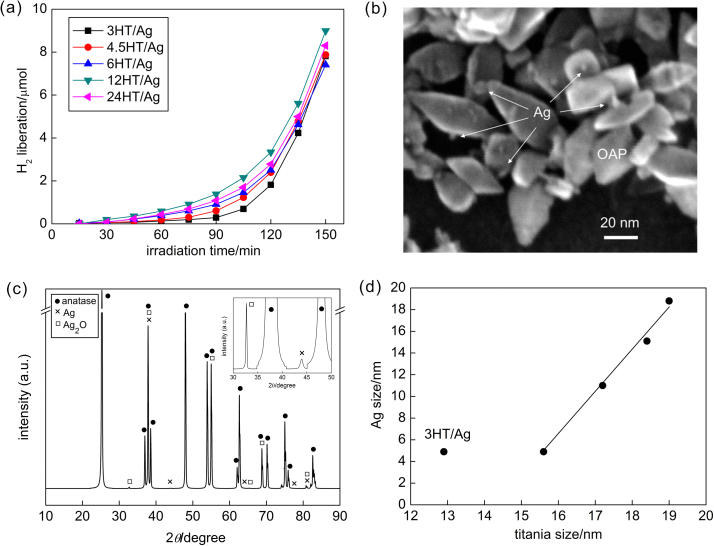
(a) H_2_ liberation during Ag deposition on OAPs, (b) STEM image of 6HT/Ag sample, (c) XRD pattern of 6HT/Ag sample, and (d) the influence of titania size on the size of silver NPs.

For preparation of silver-modified OAPs, the aqueous solution of silver nitrate (AgNO_3_) was used as metal precursor. 500 mg of each bare titania sample was used for photodeposition. The amount of silver was calculated to be 2.0 wt% to titania. Photoirradiation was carried out for 25 mL of aqueous solution of methanol (50 vol%) containing titania and AgNO_3_(aq) under magnetic stirring (500 rpm) by a 400-W high-pressure mercury lamp (Eiko-sha; light intensity of 20–22 mW) at 298 K (thermostated water bath). The process of silver deposition on OAPs was carried out for 150 min under deaerated condition, i.e., proceeded by 15-min Ar bubbling to remove oxygen from the tubes. The tubes were sealed with rubber septa and the effectiveness of deaeration was checked by gas chromatography (GC-TCD). During photoirradiation charge carriers (e•/h^+^) were formed (Eq. (1)). Holes were scavenged by methanol forming formaldehyde (Eq. (2)), while Ag^+^ ions were reduced by electrons to zero-valent Ag (Eq. (3)). Then, hydrogen was formed on deposited Ag NPs (Eq. (4); and Fig. 1a).

(1) TiO_2_ + *hν* → e• + h^+^

(2) CH_3_OH + h^+^ → HCHO + H^+^

(3) Ag^+^ + e• → Ag (during photodeposition)

(4) H^+^ + e• → 1/2 H_2_ (on photodeposited Ag NPs)

The H_2_ liberation was measured during photodeposition for every 15 min. The obtained photocatalysts were centrifuged, washed with methanol and three times with Milli-Q water and freeze dried. The codes of the samples were defined as follow: HT-duration/Ag, for example, 3HT/Ag meant OAPs-containing titania sample prepared for 3-h HT and then modified with silver.

### 2.2 Characterization of samples

The morphology of samples was characterized by scanning transmission electron microscopy (STEM, HD-2000). The surface composition and oxidation states of elements were analyzed by XPS on JEOL JPC-9010MC (MgKα X-ray) spectrometer. XRD analysis (phase content, crystallinity and crystallite size of OAP; crystallite size of Ag) was performed using a Rigaku intelligent X-ray diffraction system SmartLab equipped with a sealed tube X-ray generator (a Cu target). Photoabsorption properties were examined by diffuse reflectance spectroscopy (DRS) on a JASCO V-670. Barium sulfate and respective bare OAPs samples were used as references. Electron mobility was evaluated by time-resolved microwave conductivity (TRMC) method, as described in previous reports [39], [50], [51], under irradiation at two fixed wavelengths, i.e., at 355 nm and 545 nm. The amount of deposited silver was determined by flame atomic absorption spectroscopy (FAAS, Shimadzu AA-6200).

### 2.3 Photocatalytic activity test

The photocatalytic activity was tested for oxidative decomposition of acetic acid under UV/vis irradiation (the same irradiation system as for silver photodeposition). In each experiment, 50 mg of photocatalyst was suspended in 5 mL of aqueous acetic acid (5 vol%) and then irradiated under magnetic stirring (1000 rpm). Amounts of liberated CO_2_ in a gas phase were evaluated by gas chromatography (TCD-GC, Shimadzu GC-8A equipped with Porapak-Q column).

The visible light activity was evaluated for oxidation of 2-proponal to acetone in a sealed testing tubes of 35-mL volume. 50 mg of photocatalyst was suspended in 5 mL of 2-propanol (5 vol%) and irradiated under magnetic stirring (thermostated water bath) in a photoreactor equipped with 300-W xenon lamp (CX-04E Inotech, Japan), a cold mirror, an IR filter (water bath) and a cut-off filter Y45 (Asahi Techno Glass); with vis light intensity of 250–350 μW (lamp spectrum and the photoreactor were described in detail in previous reports [52]). The generated acetone was analyzed in liquid phase (after filtration of 0.3 mL sample through a syringeless filter, (Whatman, PVDF)) by gas chromatography (GC-FID, Shimadzu GC-14B equipped with PEG-20 M 20% Uniport B column, temperature: 80 °C, sample volume: 0.5 μL).

## 3 Results and discussion

### 3.1 Preparation and characterization of Ag/OAPs samples

**Fig. 2:**
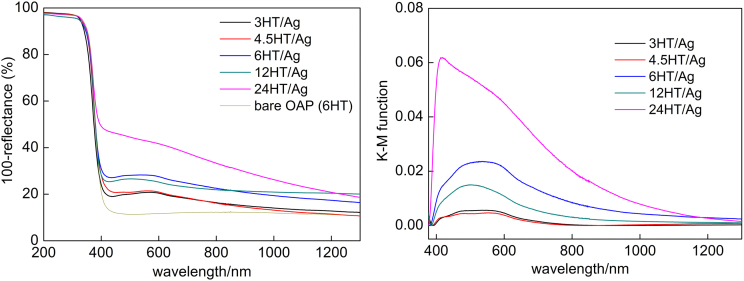
DRS spectra of (left) bare OAPs sample (6HT) and Ag-modified OAPs samples taken with BaSO_4_ as reference, and (right) Ag-modified OAPs samples taken with respective bare OAPs samples as references.

OAPs samples were prepared by varying in duration of HT (3 h, 4.5 h, 6 h, 12 h and 24 h), which resulted in preparation of products with different physicochemical properties, as shown in Table 1. It was found that prolongation of HT duration resulted in higher crystallinity, larger crystallite size, lower specific surface area and lower amount of electron traps (ET density), as described in detail in previous papers [39], [47]. In this regard, it has been expected that the properties of titania should influence the course of silver photodeposition as well as the properties of formed Ag NPs. Indeed, the properties of Ag NPs differed in all studied samples, as shown in Fig. 1, Fig. 2. However, the course of hydrogen evolution during Ag deposition did not differ significantly between samples, as shown in Fig. 1a. It is thought that low activity of silver-modified titania for hydrogen evolution (much lower than that of gold- and/or platinum-modified titania [45], [53], [54], due to (i) similar work function of silver and electron affinity of titania, and (ii) high activation overpotential for hydrogen evolution on silver) caused negligible differences in activity between all samples. It was found that formation of Ag NPs was completed during first 2 h of irradiation since after this time linear evolution of hydrogen was noticed (as shown in Fig. S1(left)). The differences in the course of hydrogen evolution during first two hours of irradiation (and the time needed for complete deposition of silver (CDT) shown in the last column of Table 1) correspond to content of electron traps (ETs), i.e., the lower the content of ETs is, the faster is reduction of silver cations (Fig. S1(right)), and thus the higher is evolution of hydrogen. For example, the lowest activity of 3HT/Ag corresponds to the larges content of ETs.

**Table 1:** Properties of bare OAPs samples, crystallite sizes of silver NPs of Ag/OAPs samples and complete deposition time (CDT).

Code	HT time/h	Crystallinity (%)^(^a^)^	Size/nm	SSA/m^2^ g^−1(^a^)^	Total OAP content (%)^(^a^)^	ET density/mmol g^-1(^a^)^	Amount of Ag/wt%	Ag size/nm (±0.05)	CDT/min
3HT/Ag	3	28	12.9	250	7	0.239	1.8	4.9	111.9
4.5HT/Ag	4.5	60	15.6	170	35	0.158	1.8	4.9	107.5
6HT/Ag	6	78	17.2	124	64	0.114	2.0	11.0	105.3
12HT/Ag	12	83	18.4	81	61	0.098	2.1	15.1	103.3
24HT/Ag	24	81	19.0	80	62	0.065	1.7	18.8	105.9

a Values measured and reported previously [39], SSA-specific surface area, CDT-complete deposition time of silver NPs on OAPs (intersection with the x-axis shown in Fig. S1(left)).

The microscopic observations confirmed the presence of silver NPs on the surface of OAPs, as shown in Fig. 1b. The XRD patterns (representative shown in Fig. 1c) confirmed anatase form of titania and silver in two crystalline forms: Ag and Ag_2_O. The presence of Ag_2_O is not surprising since silver is easily oxidized, and even when zero-valent silver is formed (under anaerobic conditions), the contact of metallic silver with air results in surface oxidation of silver [46], [53], [54], [55], [56]. The crystallite sizes of zero-valent silver are shown in Table 1. Prolongation of duration of HT reaction resulting in formation of larger anatase crystallites caused formation of larger silver NPs. The correlation between sizes of anatase and silver NPs confirms that properties of titania are crucial for the properties of resultant deposits of silver, as shown in Fig. d and Fig. S2 (the exception for 3HT/Ag sample is caused by incomplete conversion of TNWs into anatase (only 28% crystallinity) due to insufficient duration of HT reaction). Similar tendency was reported for photodeposition of gold on 15 commercial titania samples, i.e., the direct correlation between sizes of titania (anatase or rutile) and gold [57], due to the influence of crystalline defects (electron traps, ETs), which are nucleation sites for gold clusters [58]. Gold NPs of larger sizes (due to aggregation) were deposited on titania of larger sizes (better crystallinity) with lower content of ETs, while larger content of ETs in smaller titania NPs (larger SSA) induced formation of small gold nanoclusters [59]. This explanation is also valid in the present study, since an increase in the content of ETs resulted in an increase in amount of highly dispersed small NPs of silver (Fig. S2(left)).

The photoabsorption properties of Ag/OAPs samples were examined using BaSO_4_ and respective bare OAPs samples as references, and obtained data are shown in Fig. 2. The strong photoabsorption in UV range (Fig. 2(left)) is caused by band-gap excitation of anatase with absorption edge at ca. 390 nm (as clearly shown for bare sample (6HT), photoabsorption properties of other OAPs samples were reported elsewhere [39]). It should be reminded that LSPR (localized surface plasmon resonance) of silver overlaps with photoabsorption of titania at near UV region. Therefore, DRS spectra taken with respective titania as reference are more recommended in the case of Ag/TiO_2_ samples to analyze LSPR feature of silver deposits, as shown in Fig. 2(right). It is clear that OAPs samples with better morphology (prepared for longer duration of HT: 6–24 h, Table 1) modified with silver NPs exhibited more intensive coloration (darker brown-grey color), detected as more intensive photoabsorption at vis region. The 24HT/Ag sample exhibited the highest vis photoabsorption among all samples, while the photoabsorption properties of 3HT/Ag and 4.5HT/Ag samples were the poorest. It should be noted that not only the intensity of photoabsorption but also width of LSPR peak differed between samples, and those samples with the highest photoabsorption intensities had also the broader LSPR peak indicating the high polydispersity of silver deposits. It is known that the properties of plasmonic metal deposits influence the position and shape of LSPR peak [60], [61], [62], [63], and it has been suggested that the broader the LSPR is, the higher is photocatalytic activity under visible light as a result of efficient use of all emitted photons [52], [64]. Therefore, it is expected that the 24HT/Ag sample should exhibit higher vis activity than other silver-modified OAPs samples due to efficient light harvesting.

The chemical states of elements and the surface compositions were examined by XPS measurements, and obtained data are shown in Table 2 and Fig. S3. The ratio of oxygen to titanium (O:Ti) exceeded two (TiO_2_), reaching 2.2–2.8 for samples prepared for shorter HT (3–6 h) and 3.5–4.7 for longer duration of HT (12–24 h). The enrichment of the titania surface with oxygen for bare OAPs samples was explained as a result of prolongation of HT reaction necessary for efficient adsorption of water/hydroxyl groups on the titania surface [54]. The larger content of oxygen in the form of hydroxyl groups was confirmed for OAPs samples prepared for longer HT by deconvolution of oxygen peak, as shown in Table 2. For example, the content of lattice oxygen reached 56.1% for sample prepared for 3 h of HT and only 25.5% for that prepared for longest duration of HT.

**Table 2:** Surface composition of Ag/OAPs samples determined by XPS analysis, and fraction of oxidation states of Ag, Ti, O and C from deconvolution of Ag3d_5/2_, Ti 2p_3/2_, O 1s and C 1s.

Surface composition of Ag/OAPs samples and oxidation states of silver										
Samples	Content (at. %)				Ratio		Ag (at. %)	Valent state (%)		
	Ti	O	C	Ag	O/Ti	C/Ti		Ag^2+^	Ag^+^	Ag^0^
3HT/Ag	12.9	36.1	50.9	0.12	2.8	3.9	0.93	0.2	93.3	6.5
4.5HT/Ag	13.2	35.4	51.3	0.14	2.7	3.9	1.06	0.0	97.2	2.8
6HT/Ag	19.3	41.5	39.0	0.20	2.2	2.0	1.04	0.0	92.3	7.7
12HT/Ag	6.0	28.0	66.0	0.06	4.7	11.0	1.00	2.9	94.4	2.7
24HT/Ag	9.1	31.8	58.8	0.39	3.5	6.5	4.29	0.6	89.4	10.0

Fractions of oxidation states for titanium, oxygen and carbon								
Samples	Ti 2p_3/2_ (%)		O 1s (%)			C 1s (%)		
	Ti^4+^	Ti^3+^	TiO_2_	Ti-OH^a^	Ti-OH^b^	C-C	C-OH	C = O
3HT/Ag	96.3	3.7	56.1	36.6	7.3	80.4	10.3	9.3
4.5HT/Ag	98.1	1.9	53.5	38.6	7.9	71.4	19.4	9.2
6HT/Ag	99.1	0.9	68.9	26.7	4.4	74.3	16.4	9.3
12HT/Ag	99.3	0.7	32.3	40.9	26.8	66.3	23.6	10.1
24HT/Ag	100.0	0.0	25.5	49.1	25.4	71.1	15.1	13.8

Ti-OH^a^: Ti-(OH)-Ti/Ti_2_O_3_/C=O, Ti-OH^b^: Ti-OH/C-OH.

The form of carbon was similar for all silver-modified OAPs samples, where carbon 1s region, deconvoluted for three peaks, could be assigned to C–C, C–OH and C=O states with binding energies (BE) of ca. 284.8 eV, 286.2 eV and 288.7 eV, respectively, and the obtained contents were 66.3–80.4%, 10.3–23.6% and 9.2–13.8%, respectively. Carbon is always detected in titania samples and comes from the atmosphere during preparation of samples for XPS measurements [65]. Titanium existed mainly in Ti^4+^ form (96.3–100%) in all silver-modified OAPs samples. The extension of the duration of HT reaction resulted in the formation of more crystalline TiO_2_ structure with a smaller amount of oxygen vacancies, i.e., Ti^3+^ (3.7–0.0% with increase in HT duration from 3 h to 24 h), corresponding to the density of ETs (Fig. S4). The deconvolution of Ag 3d_5/2_ peak indicated that Ag^+^ was the main form of silver in all sample. However, it should be pointed that 24HT/Ag sample had the largest content of zero-valent silver (Ag^0^) among all samples (ca. 10%). This is not surprising since this sample possesses the largest silver NPs and thus oxidation of silver by air should results in oxidation of only surface of silver NPs keeping metallic character of the large silver core (Ag@Ag^+^).

### 3.2 Photocatalytic activity of Ag/OAPs samples

Photocatalytic activity was tested under UV and vis irradiation for oxidative decomposition of acetic acid and oxidation of 2-propanol, respectively. Moreover, TRMC measurements under UV and vis irradiation were performed to correlate the photocatalytic activity with mobility of charge carriers.

**Fig. 3:**
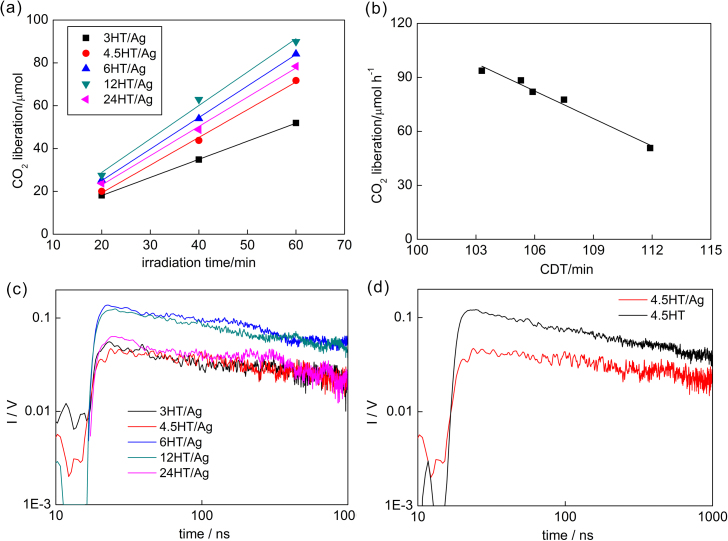
(a) CO_2_ liberation during the decomposition of acetic acid, (b) the correlation between photocatalytic activity and CDT during silver photodeposition, (c) TRMC results measured at 355 nm for silver modified samples, (d) comparison of TRMC signals between bare and silver modified sample prepared for 4.5-h HT.

The results of photocatalytic activity under UV/vis irradiation are shown in Fig. 3a. It should be pointed that silver NPs exhibited only negligible activity when deposited on insulator (silica), as shown in Fig. S5(left). The photocatalytic activity of 12HT/Ag sample was the highest, but it should be pointed that activities of all other samples did not differ significantly except for 3HT/Ag sample with the worst morphology (unreacted TNWs). Interestingly, different tendency was obtained for bare OAPs samples, where the photocatalytic activity mainly correlated with the morphology, i.e., the larger the content of OAPs in the final product was, the higher was the photocatalytic activity (the most active was 6HT sample [39], [47]). The enhancement of activity after titania modification with silver was also the highest for 12HT/Ag sample reaching ca. 2.7. It is thought that under UV irradiation the activity enhancement is mainly caused by retardation of the recombination between charge carriers, where photogenerated electrons are sinking in silver NPs, and thus formed Schottky barrier hinders the e•/h^+^ recombination. The clear correlation (Fig. 3b) between the photocatalytic activity and CDT time during silver deposition (Table 1) proves that electron transfer from titania to silver NPs is crucial for resultant photocatalytic activity. TRMC results measured under irradiation with 355 nm (Fig. 3c) showed that 6HT/Ag and 12HT/Ag samples with the highest photocatalytic activities had also the highest maximum of TRMC signal (I_max_). Unfortunately, the clear correlation between photocatalytic activity and TRMC results could not be drawn, due to complex dependences between properties, activities and electron mobility. For example, for bare OAPs samples although, the tendency between *I*_max_ and photocatalytic activity could be observed, the clear correlation could be only obtained for OAPs samples with almost the same surface properties (SSA, crystallinity, crystallite size) and only differed by morphology [39]. Interestingly, the comparison of TRMC data for bare (4.5HT) and silver-modified OAPs (4.5HT/Ag) samples indirectly confirmed the electron transfer from titania to silver NPs (decrease in *I*_max_) and higher photocatalytic activity of Ag/OAPs (slower signal decay), due to the inhibition of charge carriers’ recombination, as shown in Fig. 3d.

**Fig. 4:**
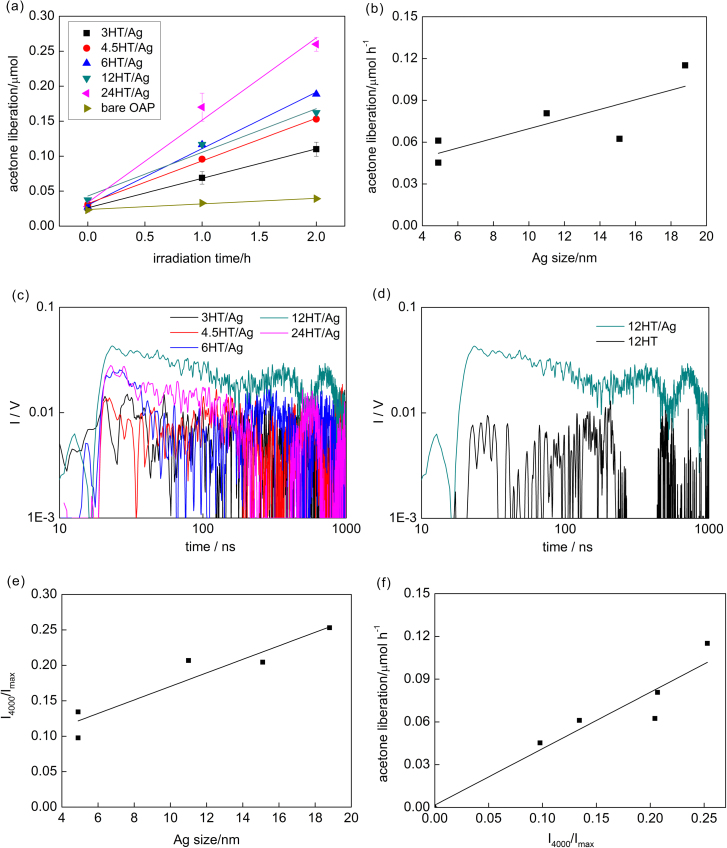
(a) Acetone liberation under visible light irradiation for bare and Ag-modified OAPs, (b) the correlation between size of silver NPs and photocatalytic activity, (c) TRMC results under excitation with 545 nm for silver-modified samples, (d) Comparison of TRMC signals for bare and silver-modified OAPs prepared for 12-h HT reaction, (e) the correlation between the decay of the TRMC signal and the size of silver NPs (f) the correlation between the decay of the TRMC signal and vis photocatalytic activity.

The photocatalytic activity under vis irradiation was tested for 2-propanol oxidation to acetone, and the obtained data are shown in Fig. 4a. It is worth to mention that similar to activity tests under UV irradiation, silver NPs supported on silica have negligible photocatalytic activity (Fig. S5(right)), confirming that the presence of semiconductor is necessary for plasmonic photocatalysts (electron transfer from silver NPs via conduction band of titania to adsorbed oxygen [52]). Bare OAPs had negligible photocatalytic activity under visible light irradiation due to wide bandgap of anatase. Whereas, the modification of OAPs with silver NPs resulted in significant enhancement of photocatalytic activity for all samples (Fig. S6). As expected, the highest photocatalytic activity was obtained for the 24HT/Ag sample with the best photoabsorption properties, i.e., the broadest and the most intense LSPR peak. The 6HT/Ag sample with slightly narrower LSPR peak exhibited also very high photocatalytic activity. The photoabsorption properties directly correlate with the properties of silver NPs, where broader LSPR means the presence of silver NPs with larger polydispersity in size/shape. Polydispersity correlates with mean size of silver NPs, i.e., the larger polydispersity is, the larger are silver NPs (mean/average size estimated by XRD). Therefore, the correlation between the size of silver NPs and the photocatalytic activity (Fig. 4b) indicates that larger sizes of silver NPs, and thus higher polydispersity of silver deposits accelerates the photocatalytic activity under vis irradiation.

TRMC measurements were carried out also under vis irradiation (545 nm), and obtained data are shown in Fig. 4c and Table S1. For the 12HT/Ag sample with the most intense TRMC signal, the comparison between bare and modified samples clearly suggests the participation of “hot” electrons in the mechanism of plasmonic excitation (transfer of “hot” electrons from plasmonically excited NPs of noble metal to conduction band of titania [40], [52], [66]) due to much higher *I*_max_ and slower decay of the signal, as shown in Fig. 4d. Recently, similar TRMC signals under vis excitation have been reported for commercial titania (P25) modified with fine gold nanoclusters (ca. 2 nm) [67]. Although, TRMC data under vis excitation showed the highest *I*_max_ for the 12HT/Ag sample with average activity, the signal decay correlated well with photocatalytic activity for all samples (Fig. 4e), and thus with the size of silver NPs (Fig. 4f). Therefore, TRMC results have confirmed the conclusions drawn from photocatalytic activity tests, i.e., that the size of silver NPs and thus the polydispersity of silver deposits were the key-factors of photocatalytic activity of silver-modified titania samples.

## 4 Summary and conclusions

Plasmonic photocatalysts composed of silver NPs and faceted anatase could be easily prepared by photodeposition method. It was found that the properties of support (OAPs) significantly influenced the resultant properties of silver NPs. Smaller silver NPs were formed on smaller OAPs with larger content of electron traps (ETs). Whereas, larger OAPs with higher crystallinity and smaller content of ETs induced formation of larger silver NPs due to silver aggregation. Therefore, it was concluded that ETs worked as nucleation sites for silver.

The modification with silver resulted in enhanced photocatalytic activity under both UV and vis irradiation. It was found that larger size of silver NPs, and thus larger polydispersity of silver deposits resulting in broad and intense LSPR peak caused enhanced vis activity due to intensified light harvesting. The photocatalytic activity correlates with TRMC data. Under UV irradiation: (i) the most active samples exhibit also the highest TRMC signal (*I*_max_), and (ii) a decrease in *I*_max_ and slower decay for silver-modified samples in comparison to bare OAPs indicate an electron transfer from titania to silver, and thus retardation of charge carriers recombination, respectively. Under vis irradiation: (i) more intense TRMC signal, observed for silver modified sample, indicates an electron transfer from plasmonically excited silver to conduction band of titania, and (ii) slower decay for the most active samples, i.e., possessing more polydispersed silver NPs with broad LSPR peak, correlates well with vis photocatalytic activity data. Therefore, TRMC data confirm that the polydispersity of silver deposits is the key-factor for vis photocatalytic activity of silver-modified titania samples.
