# Supplementary material for: Silver-modified octahedral anatase particles as plasmonic photocatalyst
Source: Catal Today. 2018 Jul 15;310:19–25. doi: 10.1016/j.cattod.2017.05.039 (PMC5946688; doi:10.1016/j.cattod.2017.05.039)
Supplement: Supplementary file 1 [file mmc1.docx]

**Electronic supplementary information**

for

**Silver modified octahedral anatase particles for plasmonic photocatalysis**

Z. Wei^a^, M. Janczarek^a,b^, M. Endo^a^, C. Colbeau-Justin^c^, B. Ohtani^a^, E. Kowalska^a^*

^a^ Institute for Catalysis, Hokkaido University, N21, W10, 001-0021 Sapporo, Japan

^b^ Department of Chemical Technology, Gdansk University of Technology, Narutowicza Str. 11/12, 80-233 Gdansk, Poland

^c^ Laboratory of Physical Chemistry, Paris-Sud University, Bat. 349, 91405 Orsay, France

*Corresponding author. Tel.: +81 117069130,

E-mail address: [kowalska@cat.hokudai.ac.jp](mailto:kowalska@cat.hokudai.ac.jp) (E. Kowalska);

Influence of titania properties on complete deposition time (CDT):


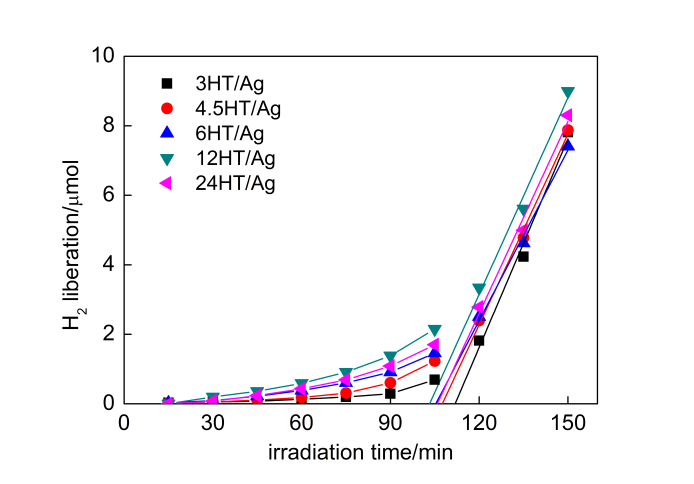

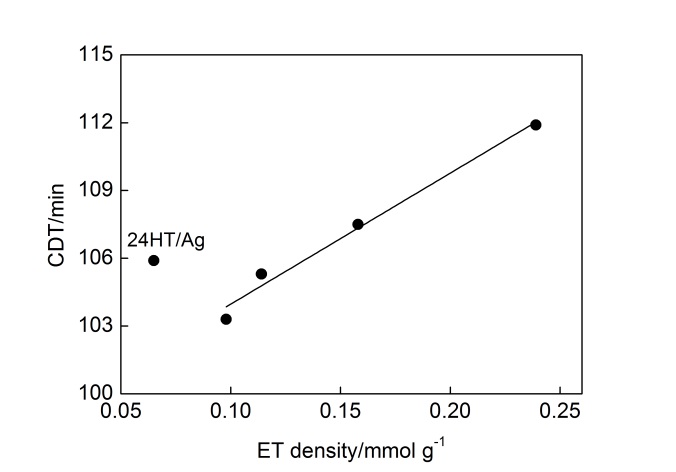


**Fig. S1.** (left) Complete deposition times (CDT) of the samples (intersection with x-axis),

(right) correlation between ET density and CDT.

Influence of titania properties on the Ag size:


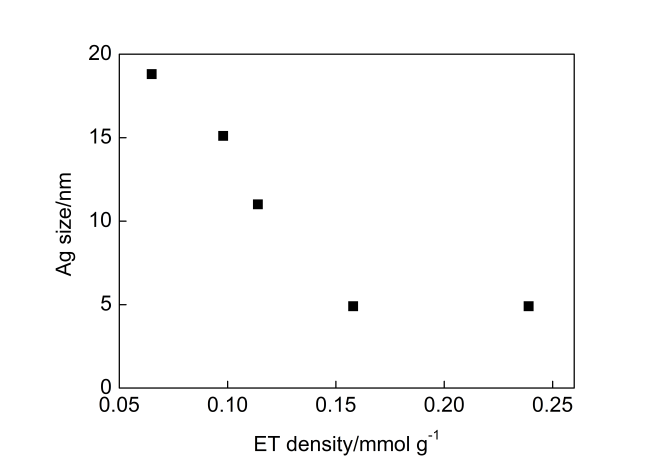

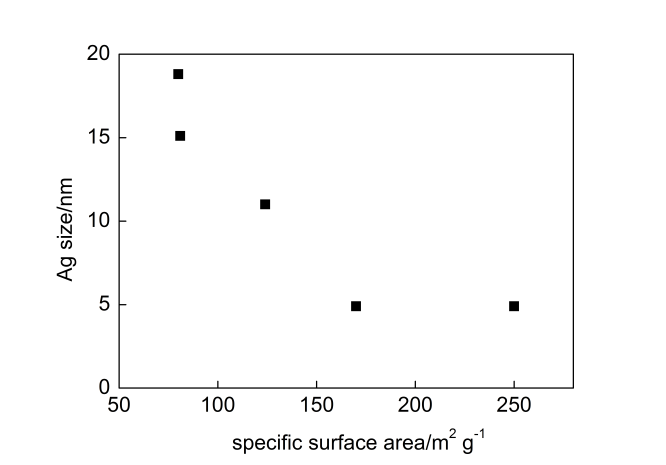


**Fig. S2.** Influence of titania properties: ET density (left) and specific surface area (right) on Ag size.


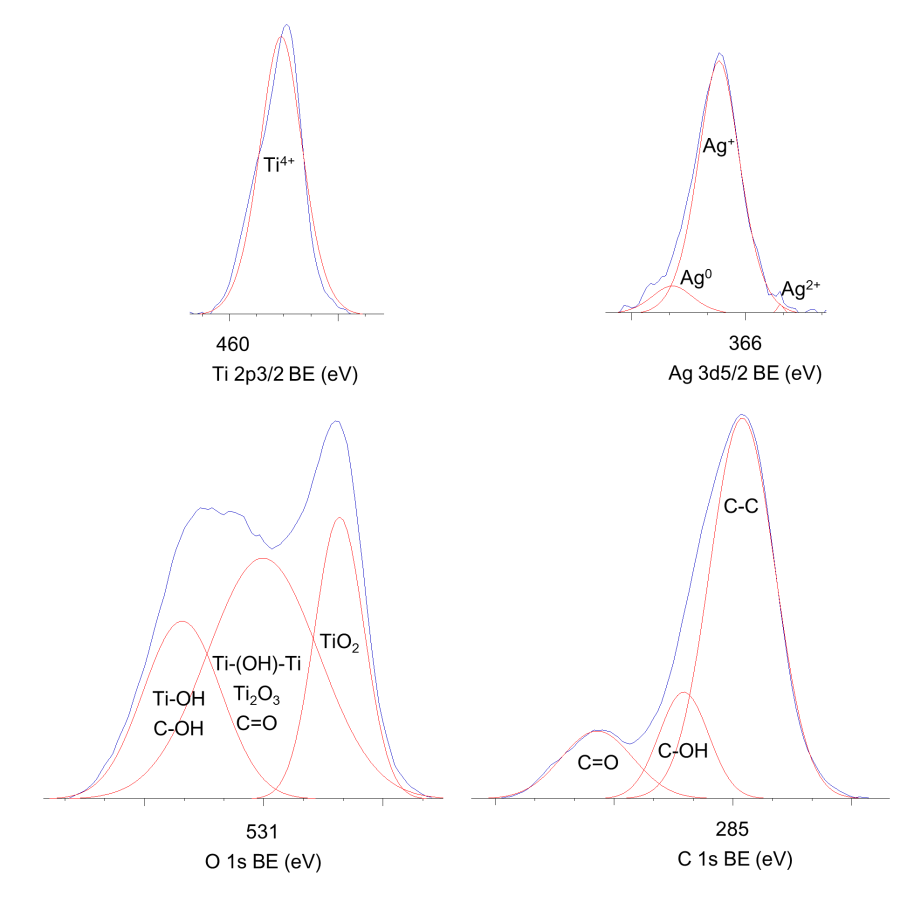


**Fig. S3.** XPS results for Ti 2p_3/2_, O 1s, C 1s and Ag 3d_5/2_ for 24HT/Ag.


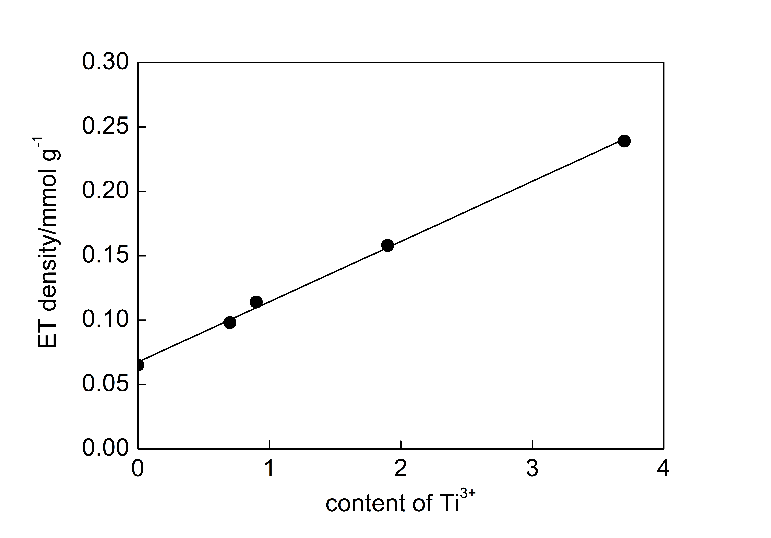


**Fig. S4.** The correlation between density of ETs and the content of Ti^3+^.

Photocatalytic activity of silver NPs deposited on silica

Silver NPs were prepared by reduction of Ag^2+^ with trisodium citrate, and then they were deposited on SiO_2_.


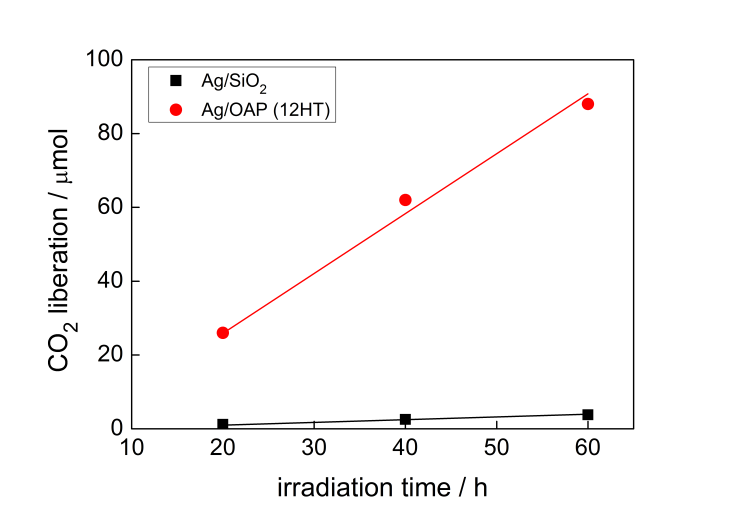

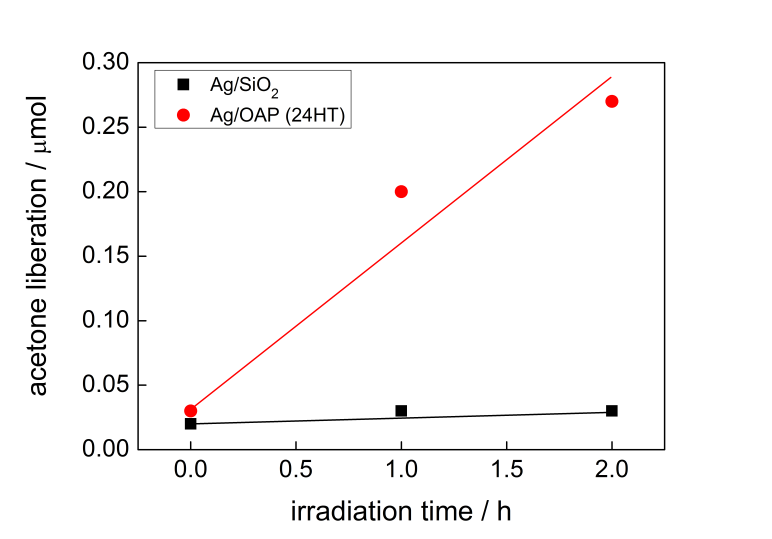


**Fig. S5.** The photocatalytic activity of Ag/SiO_2_ samples compared with the most active Ag/OAPs in two reaction systems: UV/vis photooxidation of acetic acid (left) and visible light induced oxidation of 2-propanol (right).

Influence of sample properties on the visible light activity:


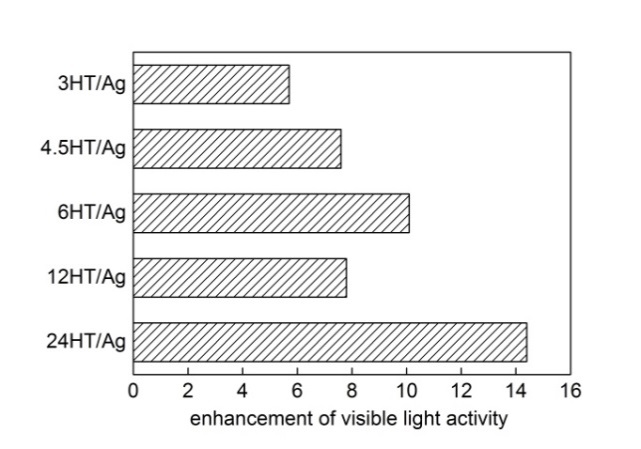


**Fig. S6.** The enhancement of visible light activity after surface modification.

**Table S1.** TRMC results

| NO. | Code | HT  time/h | measured at 355 nm | | | measured at 545 nm | | |
| --- | --- | --- | --- | --- | --- | --- | --- | --- |
|  |  |  | Max  /V | *I*_40ns_  /*I_max_* | *I*_4000ns_  /*I_max_* | Max  /V | *I*_40ns_  /*I_max_* | *I*_4000ns_  /*I_max_* |
| 1 | 3HT/Ag | 3 | 0.052 | 0.87 | 0.31 | 0.019 | 0.35 | 0.10 |
| 2 | 4.5HT/Ag | 4.5 | 0.046 | 0.87 | 0.39 | 0.022 | 0.15 | 0.13 |
| 3 | 6HT/Ag | 6 | 0.141 | 0.80 | 0.26 | 0.027 | 0.47 | 0.21 |
| 4 | 12HT/Ag | 12 | 0.128 | 0.84 | 0.26 | 0.039 | 0.79 | 0.20 |
| 5 | 24HT/Ag | 24 | 0.066 | 0.74 | 0.21 | 0.030 | 0.64 | 0.25 |
